# Supplementary material for: Changes in enzyme activity and microbial community of rhizosphere soil under continuously monocultured Passiflora edulis treatment
Source: PLoS One. 2025 Jul 16;20(7):e0328363. doi: 10.1371/journal.pone.0328363 (PMC12266385; doi:10.1371/journal.pone.0328363)
Supplement: Attachment — Submitted filename: PONE-D-24-60574 reviewed.pdf [file pone.0328363.s007.pdf]

Manuscript Number: PONE-D-24-60574

Title: Changes in Rhizosphere Enzyme Activity and Microbial Community of Continuously Cultivated *Passiflora edulis.*

Overview and general recommendation:

This subject is important because the current study analyzed the main physicochemical properties, phenolic acid content, and microbial community changes in the rhizosphere soil of passion fruit under different continuous cropping durations.

I found the manuscript well written, well described and the references were approximately new. I listed **minor points** below for editing.

Comments to Authors:

Minor points

1- In abstract, 14line and 18line: You wrote (We 2 times)! The rule of manuscript writing is to avoid using (We). So you should delete (**We**) and use **formal scientific words** (**This study or The current study or The present study**).

2- After abstract; you should write **Key words** of your manuscript, so why you did not write them!!!

3- In introduction, the rule of manuscript writing is to write the aims of your study in the end of introduction part. Kindly add the aims.

4- In 371line: You wrote **discuss!!!** Kindly convert it to **discussion**.

5- In discussion, 382line, 404line and 425line: You wrote (Our 3 times)! The rule of manuscript writing is to avoid using (Our). So you should delete (Our), use the **formal scientific words** (**This study or The current study or The present study**).

Kind regards
