# Supplementary material for: Changes in enzyme activity and microbial community of rhizosphere soil under continuously monocultured Passiflora edulis treatment
Source: PLoS One. 2025 Jul 16;20(7):e0328363. doi: 10.1371/journal.pone.0328363 (PMC12266385; doi:10.1371/journal.pone.0328363)
Supplement: Attachment — Submitted filename: Response to Editor-0515.docx [file pone.0328363.s010.docx]

Dear editor:

Thank you very much for your valuable feedback on this article, such as:

1. Please amend the title either on the online submission form or in your so that they are identical.

2. Please ensure that you refer to Figure 6 in your text as, if accepted, production will need this reference to link the reader to the figure.

We sincerely thank the editorial department and experts for their valuable feedback: in this revised version, all changes made to the original manuscript have been incorporated into the document using revision mode. Below this letter is a list of point by point explanations and responses to the editor and reviewers：

1.have made corresponding modifications to question 1.

2. I have made full text modifications to the link to the figure and table for question 2.

Sincerely

Weiwei Lin
