# Supplementary material for: Changes in enzyme activity and microbial community of rhizosphere soil under continuously monocultured Passiflora edulis treatment
Source: PLoS One. 2025 Jul 16;20(7):e0328363. doi: 10.1371/journal.pone.0328363 (PMC12266385; doi:10.1371/journal.pone.0328363)
Supplement: Attachment — Submitted filename: Response to Editor-0612.docx [file pone.0328363.s011.docx]

Dear Editor：

We appreciate the reviewer’s comments on standardizing the manuscript formatting and submitting sequencing data.

All formatting inconsistencies throughout the manuscript (including referencing styles, figure/table captions, and text spacing) have been carefully revised to adhere to PLOS ONE’s guidelines.

The required sequencing data have been deposited in the National Center for Biotechnology Information (NCBI) under the accession number [PRJNA1280495]. The detailed data can be accessed via the following link: [https://dataview.ncbi.nlm.nih.gov/object/PRJNA1280495?reviewer=i1ut7ml58v5vpt2bdq41qqfogb].

These revisions ensure compliance with the journal’s standards and enhance the transparency and reproducibility of the study. We thank the reviewer for prompting these improvements.

Sincerely

Weiwei Lin
